# Ein integriertes Konzept für nachhaltige hybride Arbeit – Erkenntnisse und Handlungsempfehlungen aus einem Transformationsprojekt

**DOI:** 10.1365/s40702-022-00882-9

**Published:** 2022-05-31

**Authors:** Thomas Kreuzer, Julia Lanzl, Jörg Römmelt, Manfred Schoch, Simon Wenninger

**Affiliations:** 1 Institutsteil Wirtschaftsinformatik, Fraunhofer FIT, Alter Postweg 101, 86159 Augsburg, Deutschland; 2 Lehrstuhl für Digitales Management, Universität Hohenheim, Schloss Osthof-Ost, 70599 Stuttgart, Deutschland; 3 Kernkompetenzzentrum Finanz- & Informationsmanagement, Hochschule Augsburg, Institutsteil Wirtschaftsinformatik, Fraunhofer FIT, Alter Postweg 101, 86159 Augsburg, Deutschland; 4 Kernkompetenzzentrum Finanz- & Informationsmanagement, Universität Augsburg, Alter Postweg 101, 86159 Augsburg, Deutschland

**Keywords:** New Work, Hybride Arbeit, Nachhaltigkeit, Good Practices, Digitale Transformation, COVID-19, New Work, Hybrid Work, Sustainability, Good Practices, Digital Transformation, COVID-19

## Abstract

Große gesellschaftliche Herausforderungen wie die COVID-19-Pandemie und der globale Klimawandel haben die Art und Weise wie Menschen zusammenarbeiten und über Arbeit nachdenken für immer verändert. Aber was bedeutet das konkret für Organisationen? Genau diese Frage wird in diesem Artikel anhand einer Fallstudie über das Transformationsprojekt eines deutschen Forschungsinstituts hin zu nachhaltiger hybrider Arbeit untersucht. Der analysierte Fall ist besonders interessant, weil sich das Forschungsinstitut zusätzlich der Herausforderung eines anstehenden Umzugs noch während der COVID-19-Pandemie gegenübersah. Der Artikel zielt darauf ab, Erfahrungen und Handlungsempfehlungen für eine adäquate Gestaltung der Rahmenbedingungen für nachhaltige hybride Arbeit in allen Phasen der Transformation darzustellen. Erstens wird ein konkretes New-Work-Konzept entlang mehrerer Dimensionen für nachhaltige hybride Arbeit präsentiert, das durch Erkenntnisse in der Literatur und durch Erfahrungen aus deren praktischer Anwendung entwickelt wurde. Zweitens werden Einblicke in die Umsetzung gegeben, dabei das Zusammenspiel mehrerer interdisziplinärer Teams aufgezeigt und Good Practices auf dem Weg zu nachhaltiger hybrider Arbeit abgeleitet. Der Beitrag hebt sich von bisherigen Erkenntnissen ab, da er hybride Arbeit und Nachhaltigkeit vereint und Wege der integrierten, interdisziplinären Umsetzung aufzeigt. So geben unsere Ergebnisse anderen New-Work-Forschenden Grundlage und Struktur, um das Konzept nachhaltige hybride Arbeit und der Entwicklung und Messung ihrer Erfolgsfaktoren tiefergehend zu untersuchen. Praktiker können das Konzept und die Good Practices als Blaupause für die eigene Transformation hin zu nachhaltiger hybrider Arbeit verwenden und davon ausgehend organisationsindividuelle Anpassungen vornehmen.

## Abstract (en)

Major societal challenges like the COVID 19 pandemic and global climate change have forever altered the way people work together and think about work. But what does this mean for organizations? This article explores this through a case study of a German research institute’s transformation project towards sustainable hybrid work. The case at hand is particularly interesting as the research institute faced the additional challenge of a pending move during the COVID 19 pandemic. The article aims to present experiences and recommendations for action for an adequate design of sustainable hybrid work in all phases of the transformation. First, it presents a concrete New Work concept along several dimensions for sustainable hybrid work, which has been developed drawing from literature and practical experiences. Second, this article provides insights into the implementation, showing the collaboration of several interdisciplinary teams and deriving good practices on the way towards sustainable hybrid work. This article complements previous findings by combining hybrid work and sustainability and showing ways of integrated, interdisciplinary implementation. Thus, our findings provide a basis and structure for other researchers to explore the concept of sustainable hybrid work and the development and measurement of its success factors in more depth. Practitioners can use the concept and the good practices as a blueprint for their own transformation towards sustainable hybrid work and decide on corresponding organizational adaptations.

## Herausforderung hybride Arbeit – Transformationsdruck in Organisationen

Die COVID-19-Pandemie und der damit verbundene deutlich gestiegene Anteil an Arbeit im Homeoffice hat zu großen Veränderungen im Arbeitsalltag geführt (Möhring et al. 2020). Forschung und Praxis sind sich inzwischen einig, dass Arbeit nach der COVID-19-Pandemie nicht mehr wie davor aussehen wird (z. B. Fayard et al. 2021; Flüter-Hoffmann und Stettes 2022). Erste Organisationen haben darauf reagiert und verkleinern ihre Büroflächen oder gestalten diese um, um den künftigen Anforderungen und Arbeitsbedingungen gerecht werden zu können (z. B. Radomsky 2022).

Ein Treiber dieser veränderten Welt nach der Pandemie ist die Erfahrung, dass Arbeit im Homeoffice effizient und produktiv sein kann. Hinzu kommen die zunehmend veränderten Erwartungen der Arbeitnehmenden an ihre Arbeitgeber, die mit Blick auf den Fachkräftemangel verstärkt in den Fokus genommen werden müssen (Hofmann et al. 2021). In den USA, aber auch in Deutschland, hat sich im Laufe der COVID-19-Pandemie bereits eine regelrechte Kündigungswelle – auch „the great resignation“ genannt – entwickelt. Gründe hierfür waren unter anderem veränderte Prioritäten bei der Auswahl des richtigen Arbeitgebers, wobei örtlich wie zeitlich flexible Arbeitsmöglichkeiten, Autonomie bei der Arbeitsgestaltung sowie die Berücksichtigung von mentaler Gesundheit durch den Arbeitgeber nun eine bedeutende Rolle spielen (Mercer 2021). In die gleiche Richtung geht der vom Philosophen Frithjof Bergmann geprägte Begriff von „New Work“, der sich auf eine selbstbestimmte und flexible Arbeit, die Mitarbeitende wirklich wollen, bezieht (Bergmann 2020). Darüber hinaus steigen die Anforderungen an die Nachhaltigkeit von Organisationen, um den menschengemachten Klimawandel zu stoppen. Dies wird von Organisationen nicht nur aufgrund ihrer gesellschaftlichen Verantwortung gefordert, sondern ist zunehmend auch für die Gewinnung und Bindung von Arbeitnehmenden relevant (Davis-Peccoud 2013). Neben der Möglichkeit, flexibel zu arbeiten, spielen bei der Arbeitssuche Aspekte der Corporate Social Responsibilty und vor allem der Nachhaltigkeit eine wichtige Rolle (Genders 2021; Harrach et al. 2014). So steigert nachhaltiges Wirtschaften in den drei Dimensionen Ökologie, Ökonomie und Soziales von der Arbeitsplatzgestaltung bis hin zur nachhaltigen Energieversorgung die Attraktivität einer Organisation unter Arbeitssuchenden und Mitarbeitenden.

**Abb. 1:**
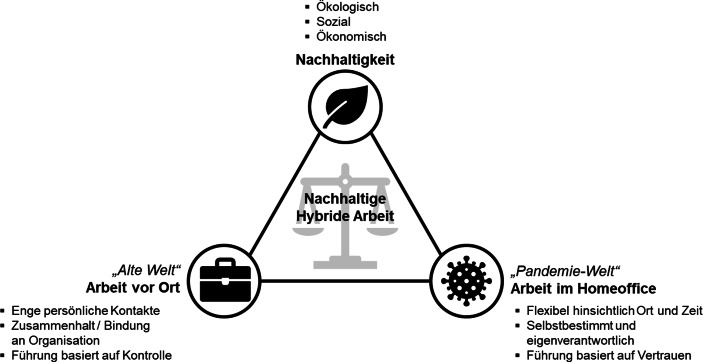
Der Dreiklang von nachhaltiger hybrider Arbeit

Anforderungen an die künftige Arbeitsgestaltung kommen daher insbesondere aus drei Richtungen (vgl. Abb. 1): Es sind vor allem die beiden vermeintlichen Gegenpole der „alten Welt“ und der „Pandemie-Welt“ zu nennen. Auf der einen Seite möchten viele Organisationen die Vorteile von Homeoffice, wie Flexibilität und Selbstbestimmtheit, auch weiterhin nutzen. Auf der anderen Seite bringt die alte Arbeitswelt („vor Ort“) Vorteile mit sich, wie beispielsweise der persönliche Kontakt zu Kolleg:innen und die dadurch verbesserte Kommunikation und Zusammenarbeit (Flüter-Hoffmann und Stettes 2022).

Die Balance zwischen diesen veränderten Erwartungen und Anforderungen zu finden, stellt Organisationen vor große Herausforderungen. Während Arbeit in Zeiten der COVID-19-Pandemie fast ausschließlich im Homeoffice und zuvor fast ausschließlich vor Ort erfolgte, wird nach der Pandemie eine Mischung aus Arbeit im Homeoffice und vor Ort erwartet, um die verschiedenen Anforderungen in Einklang zu bringen (Flüter-Hoffmann und Stettes 2022). Daraus folgende Ansprüche an Organisationen sind oft komplex, voneinander abhängig und schwierig zu überblicken.

Organisationen stellen sich daher die Frage: Wie sieht eine geeignete Arbeitsgestaltung aus, in der Vorteile der Arbeit vor Ort, Vorteile digitaler Arbeit sowie Nachhaltigkeitsaspekte berücksichtigt werden? Der Handlungsspielraum suggeriert das System einer Waage (vgl. Abb. 1). Organisationen müssen eine Antwort darauf finden, inwiefern Elemente aus der Arbeitswelt vor der COVID-19-Pandemie mit Elementen aus der Zeit der Pandemie, welche sich als erfolgreich herausgestellt haben, und ein verstärkter Fokus auf Nachhaltigkeit miteinander in Einklang bringen lassen.

Jedoch reicht es nicht, Arbeit vor Ort, Arbeit im Homeoffice und Nachhaltigkeitsinitiativen isoliert voneinander umzusetzen. Es braucht vielmehr ein Ineinandergreifen der verschiedenen Elemente hin zu einer neuen, nachhaltigen hybriden Arbeit. Fayard et al. (2021) beispielsweise nennen die Beobachtung, dass Online-Meetings im Homeoffice meist sehr aufgabenorientiert und damit zwar effizient sind, der zwischenmenschliche Zusammenhalt dadurch jedoch zu kurz kommt. Eine Studie mit 60.000 Microsoft Arbeitnehmenden hat bestätigt, dass Mitarbeitende durch die Arbeit im Homeoffice mit weniger Personen kollaborieren und sich zunehmend Silos zwischen Abteilungen bilden (Yang et al. 2022). Gerade daher hat das Büro als Ort der persönlichen Begegnung auch in Zukunft eine wichtige Rolle. Gleichzeitig reicht es nicht, lediglich die Räumlichkeiten vor Ort für den persönlichen Kontakt und Mitarbeitende in Homeoffice mit digitalen Tools auszustatten. Vielmehr müssen auch Leitplanken, Richtlinien und Strategien für die Zusammenarbeit im New Normal erarbeitet werden sowie neue Führungskompetenzen aufgebaut werden (Reibetanz et al. 2021). Beispielsweise ist das Büro vor Ort nur dann als Begegnungsstätte nützlich, wenn auch die Arbeitnehmenden dort zusammenkommen, bei denen der Austausch für die Aufgaben vorteilhaft ist. Dies bedarf einer entsprechenden Koordination.

Es braucht also eine durchdachte Transformation hin zu New Work, bei der Räumlichkeiten vor Ort so gestaltet werden, dass sie die Defizite der Arbeit im Homeoffice ausgleichen und gleichzeitig dem Wunsch einer nachhaltigen Arbeitsumgebung nachkommen. Bisher gibt es wenige wissenschaftlichen Erkenntnisse dazu, wie man diese Transformation umsetzt und was dabei beachtet werden muss. Dieser Beitrag nutz daher eine Fallstudie über das Transformationsprojekt eines deutschen Forschungsinstituts, um aufzuzeigen, wie wissenschaftliche Erkenntnisse auf dem Gebiet von New Work mit der Entwicklung und Umsetzung eines integrierten Konzepts nachhaltiger hybrider Arbeit kombiniert werden können. Als Teil des Projektteams haben die Autoren Daten aus erster Hand während der Transformation erhoben, retroperspektiv analysiert und synthetisieren die Erkenntnisse im Rahmen dieser Fallstudie. Mit der Diskussion der Erfahrungen werden übergreifende Handlungsempfehlungen für andere Organisationen abgeleitet, welche in diesem Artikel präsentiert werden.

## Das Transformationsprojekt eines mittelständischen Forschungsinstituts

Im vorliegenden Fall wird die Entwicklung und Umsetzung eines integrierten Konzepts für die Gestaltung nachhaltiger hybrider Arbeit in einer mittelständischen Organisation, mit Fokus auf die adäquate Neugestaltung des physischen Arbeitsortes sowie das dazugehörige Transformationsprojekt betrachtet. Als anwendungsorientiertes Forschungsinstitut in der Wirtschaftsinformatik beschäftigt sich die Organisation mit der Digitalisierung von Wirtschaft und Gesellschaft. Die Mitarbeitenden haben eine hohe Affinität im Umgang mit digitalen Technologien. Bis zum Ausbruch der COVID-19-Pandemie im März 2020 setzte die Organisation auf ein Arbeitsmodell, das von physischer Anwesenheit in den Büroräumen und engem Kontakt zwischen Mitarbeitenden geprägt war. Viele Mitarbeitende arbeiten in Vollzeit und waren festen Büroarbeitsplätzen zugewiesen. Teilzeitmitarbeitende nutzten dagegen offene Arbeitsbereiche. Auch wenn Homeoffice nur selektiv in Anspruch genommen wurde, ermöglichte die vorhandene IT-Infrastruktur grundsätzlich eine virtuelle Zusammenarbeit (z. B. durch mobile Endgeräte, und Kommunikations- und Kollaborationstechnologie, wie Microsoft Teams). Der Übergang auf ein größtenteils virtuelles Arbeitsmodell während der COVID-19-Pandemie war aus technischer Perspektive daher ohne größere Probleme möglich. Prozesse, die bisher physische Anwesenheit erforderten, wurden im Laufe der ersten Monate der Pandemie so weit möglich digitalisiert. Zudem rief die Organisation mehrere Initiativen ins Leben, mit Hilfe derer die pandemiebedingte (Zusammen‑)Arbeit unterstützt und verbesserte werden sollte. So wurden zum Beispiel mehrere digitale Kollaborationstools eingeführt und erweitert, Best Practices für das Onboarding neuer Mitarbeitender erarbeitet, Anlaufstellen für pandemiebedingte psychische Belastung zur Verfügung gestellt und eine virtuelle Kaffeeküche sowie Events für informellen Austausch ins Leben gerufen.

Vor diesem Hintergrund gab es für das Forschungsinstitut mehrere Gründe, das Transformationsprojekt hin zu nachhaltiger hybrider Arbeit zu initiieren. Erstens stieg unter den Mitarbeitenden das Bewusstsein dafür, dass virtuelle Zusammenarbeit zwar neuen Regeln folgt, aber auch Vorteile für bestimmte Arbeitsformen, z. B. der konzentrierten Arbeit, mit sich bringt. Physische Zusammenarbeit eignete sich dazu komplementär für kreative, gemeinsame Tätigkeiten und informellen Austausch. Grundlegende Annahme für das Transformationsprojekt war daher, dass die Mitarbeitenden zukünftig hybride Arbeit präferieren. Dies wurde im ersten Schritt des Projekts mit einer Befragung evaluiert. Zweitens war Anfang 2021 klar, dass noch im selben Jahr ein Umzug in neue Büroräumlichkeiten bevorstand. Dadurch ergab sich die Chance, das neue Arbeitsmodell ganzheitlich zu betrachten und auch die Raumgestaltung, -aufteilung, und IT-Infrastruktur von Anfang in Einklang zu bringen und zu gestalten. Drittens rückte das Thema Nachhaltigkeit zunehmend in den Mittelpunkt der Organisation. So hat das Institut einen zentralen Forschungsschwerpunkt rund um Energieversorgung, Sektorenkopplung und Mobilität und von Mitarbeitenden wurde eine Arbeitsgruppe für ökologische Nachhaltigkeit im Arbeitsalltag gegründet. Daher sollten Nachhaltigkeitsaspekte von Anfang an eine zentrale Rolle im Projekt einnehmen.

Für das Transformationsprojekt wurde ein Projektteam ins Leben gerufen, das den Prozess entlang der in Tab. 1 dargestellten vier Phasen begleitet.

**Tab. 1:** Die vier Phasen des Transformationsprojekts

	Ziele	Zentrale Aktivitäten
*Phase 1* **Bedarfserhebung**	Schaffung eines einheitlichen Verständnisses über die Erwartungen und Anforderungen aller Mitarbeitenden	Workshop auf Geschäftsführungsebene („Top-down“)
		Mitarbeitendenbefragung zum Arbeitsmodell („Bottom-up“): Beschreibung Status Quo, Erwartungen, Bedenken, Vorschläge, Best Practices
*Phase 2* **Konzeption**	Entwicklung eines organisationsindividuellen Zielbilds für nachhaltige hybride Arbeit und eines Umsetzungskonzepts	Definition und Spezifizierung von vier Dimensionen: Hybride Arbeitswelt, Nachhaltige Arbeitswelt, Infrastruktur, Kommunikation und Change
		Integrierte Umsetzungskonzeption entlang der vier Dimensionen, z. B. Entwicklung eines Raumkonzept und Aufbau eines gemeinsamen Werteverständnisses für die künftige Zusammenarbeit
*Phase 3* **Umsetzung**	Operationalisierung des Umsetzungskonzepts	Umsetzung aller entwickelten Konzepte und Maßnahmen im Rahmen des Umzugs
		Kontinuierliche Kommunikation zwischen Stakeholdern und mit allen Mitarbeitenden
*Phase 4* **Monitoring**	Evaluation aller entwickelter Konzepte und Maßnahmen	Offene, organisationsweite Diskussion zur Sammlung von Verbesserungsideen
		Kontinuierliches Monitoring der umgesetzten Maßnahmen

## Auf dem Weg zum Büro der Zukunft für erfolgreiche hybride Arbeit

Ausgehend von der Mitarbeitendenbefragung und vorhandener Erkenntnisse aus der Literatur, wurde ein Konzept erarbeitet, das verschiedene Dimensionen nachhaltiger hybrider Arbeit berücksichtigt.

Eine wichtige Dimension ist die Förderung von sozialem Miteinander (*sozialer Anker*). Die darauf abzielenden Maßnahmen fördern die sozialen Beziehungen und emotionalen Verbindungen, deren Aufbau und Pflege in der virtuellen Welt deutlich erschwert wird (Fayard et al. 2021). Dazu wurden großräumige, einladende Räumlichkeiten mit Sofaecken, Kaffeeküchen und Freizeitmöglichkeiten geschaffen. Diese sollen zum Verweilen einladen und die Möglichkeit für spontane Begegnungen und informellen Austausch liefern.

Gleichzeitig soll die Arbeit vor Ort kreative Aspekte fördern, da insbesondere unstrukturierte Kollaboration von der persönlichen Zusammenarbeit stark profitiert (Fayard et al. 2021). Die Büroflächen der Zukunft sollen daher ein *Knotenpunkt für Kollaboration* werden und über eine entsprechende Ausstattung Aspekte der Zusammenarbeit fördern. Dafür wurden sowohl offen zugängliche als auch räumlich abgetrennte Bereiche eingeplant, die über großflächige Whiteboards, Beamer, modulare Sofas und Hochtische verfügen.

Zusätzlich sind auch in der neuen Arbeitswelt Rückzugsorte für konzentrierte Arbeit notwendig. Forschung weist außerdem darauf hin, dass insbesondere neue Mitarbeitende davon profitieren, in einem bekannten Umfeld Arbeitsprozesse und Normen kennenzulernen, sowie unkompliziert Rückfragen stellen zu können. Das Büro soll also auch als Ort des Wissensaustausches dienen (Fayard et al. 2021). In Kontrast dazu steht, dass flexible Arbeit auch flexible Raumbelegungen erfordert. Eine anonyme, flexible Zuordnung der Bildschirmarbeitsplätze ermöglicht dabei eine hohe Kapazitätsauslastung und letztlich effiziente Flächennutzung. Daher gibt es grundsätzlich ein Spektrum an Möglichkeiten, Arbeitsplätze zu gestalten und zuzuweisen: von vollständiger Flexibilität (ohne Platzzuordnung) bis hin zu vollständig personalisierten und festen Bildschirmarbeitsplätzen. Organisationen müssen entscheiden, wo sie sich in diesem Spektrum sehen. Im vorliegenden Fall wurden Büros geschaffen, denen feste Teams zugeordnet sind, die die Raumkapazität bei vollständiger Nutzung aber überschreiten würden („Überbelegung“). Diese Überbelegung ist deshalb in Ordnung, da mit einem weiterhin hohen Anteil an Homeoffice gerechnet wird. Zusätzlich gibt es spezielle Projekträume, auf die ausgewichen werden kann, falls alle Teammitglieder parallel vor Ort sind. Dadurch haben Mitarbeitende Rückzugsmöglichkeiten mit eigenen Schränken und gleichzeitig die Möglichkeit zur teaminternen Kommunikation innerhalb des Büros.

Selbstverständlich ist ein wichtiger Wunsch der Belegschaft, der sich mit aktuellen Eindrücken und Umfragen aus Wissenschaft und Praxis deckt, dass weiterhin flexible Arbeit und dadurch auch die Möglichkeit der Arbeit aus dem Homeoffice besteht. Dieser Umstand führt dazu, dass zukünftig häufig die Notwendigkeit bestehen wird, zwischen der virtuellen und der physischen Arbeitswelt zu kommunizieren. Damit die Vorteile des Büros vor Ort hierdurch nicht zunichte gemacht werden (z. B. weil sich auch Mitarbeitende vor Ort vom eigenen Laptop und eigenen Büro aus einwählen), bedarf es Infrastruktur, die für die hybride Zusammenarbeit gerüstet ist. Im konkreten Fall wird dies unter anderem durch digitale Whiteboards mit Übertragungsmöglichkeit sowie moderne Videokonferenzanlagen ermöglicht.

In einer Miterarbeitendenumfrage wurde der Wunsch nach nachhaltigen Arbeitsbedingungen betont. Dies deckt sich mit gesellschaftlichen Trends, dass Mitarbeitende mehr Sinn in ihrer Arbeit sehen möchten. Hinsichtlich der Nachhaltigkeitsaspekte wurden im vorliegenden Fall die Säulen der sozialen und ökologischen Nachhaltigkeit daher explizit mitgedacht. Auf Seiten der Büroinfrastruktur wurden unter anderem höhenverstellbare Tische, begrünte offene Bereiche und Möglichkeiten zur sozialen Interaktion geschaffen. Es wurde eigene PV-Anlage installiert, die sowohl Büroinfrastruktur als auch Mobilitätsmöglichkeiten mit grünem Strom versorgt. Über Infomonitore werden die Mitarbeitenden über die jeweilige Erzeugung und den Verbrauch informiert. Gleichzeitig wurden E‑Dienstwägen angeschafft, die den Mitarbeitenden für betriebliche Zwecke zur Verfügung stehen, und E‑Fahrräder, die auch darüber hinaus gebucht und genutzt werden können.

Zuletzt wurde im Projekt festgehalten, dass eine detaillierte und persönliche Kommunikation des Konzepts, der Umsetzung und der Begebenheiten vor Ort von hoher Bedeutung ist. Insbesondere vor dem Hintergrund, dass viele Organisationen aktuell Maßnahmen zu New Work durchführen, aber noch wenig klare Erkenntnisse und Best Practices vorliegen, wurde die Partizipation der Mitarbeitenden und unkomplizierte Feedbackkanäle etabliert. Tab. 2 fasst die Kernelemente des Konzepts zusammen und beschreibt deren Umsetzung.

**Tab. 2:** Konzept für nachhaltige hybride Arbeit

Dimension		Anforderung	Erläuterung	Umsetzung in Fallstudie
*Hybride Arbeitswelt*	*Sozialer Anker*	Gemeinschaft und Aufenthalt	Ort für informellen Austausch und Zusammensein	Offene Bereiche mit Sitzmöglichkeiten und Zugang zu Kaffeeküchen etc.
		Veranstaltungen und Events	Möglichkeit Veranstaltungen in Präsenz als auch in hybrider Form abzuhalten	Abtrennbarer Raum angrenzend an offenen Bereich, der Möglichkeit für große Eventfläche bietet
	*Knotenpunkt für Kollaboration*	Zusammenarbeit und Kollaboration	Möglichkeiten zur gemeinsamen kreativen (Projekt)arbeit	Offene und geschlossene Bereiche, die (digitale und analoge) Whiteboards, Sitzmöglichkeiten, Hochtische und kreatives Material zur Verfügung stellen
	*Büro als Ort des Wissensaustauschs*	Individuelle Arbeitsplätze und Teamarbeit	Möglichkeiten zur konzentrierten Arbeit, Teamarbeit und direkten Austausch vor Ort, gerade auch für neue Mitarbeitende	Desksharing innerhalb fest zugewiesener Büros mit zusätzlichen Team- und Projekträumen
	*Brücke zwischen Arbeitswelten*	Konferenz- und Kommunikationsmöglichkeiten	Ort für den teamübergreifenden und -internen Austausch. Sowohl vor Ort als auch hybrid	Diverse Konferenzräume unterschiedlicher Größe mit Videotelefonie. Zusätzlich Büros des oberen Managements mit entsprechender IT-Infrastruktur ausgestattet
*Nachhaltige Arbeitswelt*		Nachhaltige Energieversorgung	Notwendige Infrastruktur, die nachhaltige Energieversorgung und Mobilität ermöglicht	Eigene PV-Anlage für erneuerbare Energie, Ladeinfrastruktur für Elektromobilität (Rad und Auto), sowie intelligentes Energiemanagement und Batteriespeicher
		Nachhaltige Büroinfrastruktur	Büro-Infrastruktur, die soziale Nachhaltigkeit berücksichtigt	Höhenverstellbare Tische, standardisierte und rückwärtskompatible IT-Hardware
*Querschnittsthemen*		IT-Infrastruktur	Notwendige Infrastruktur und Bandbreiten, die hybrides Arbeiten unterstützen	Diverse Video-Konferenzsysteme, mobile und standardisierte IT-Infrastruktur, digitale Whiteboards
		Kommunikationsstrategie und Change-Management	Notwendigkeit die neuen Ideen und Konzepte allen Mitarbeitenden näher zu bringen	Interaktive (virtuelle) Infoveranstaltungen und Rundgänge, die das Konzept erläutern, Fragen beantworten und Input mitnehmen

## Umsetzung und Monitoring des Konzepts

Die interdisziplinäre Projektorganisation zur Entwicklung und Umsetzung eines Konzepts für nachhaltige hybride Arbeit setzte sich aus fünf untergeordneten Teams zusammen. Drei Teams wurden aus Mitgliedern regulär vorhandener Verwaltungseinheiten gebildet: Facility-Management, IT und Human Resources (HR). Zwei weitere organisationsweite Expert:innenteams zu den Themen New Work und Nachhaltigkeit waren ebenso beteiligt. Tab. 3 zeigt die individuellen Ziele und Aufgaben der fünf Teams.

**Tab. 3:** Operative Projektorganisation

Team	Ziele	Aufgaben
*New Work*	Zufriedenheit der Mitarbeitenden	Entwicklung Konzept für nachhaltige hybride Arbeit
	Attraktivität als Arbeitgeber	Detailplanung der Konzeptumsetzung
	Effizientes Arbeiten	Ausarbeitung Kommunikationsstrategie
	Nutzung der Räumlichkeiten	Steuerung und Abstimmung mit Stakeholdern
*Facility-Management*	Flexibel nutzbare Räumlichkeiten, die alle Anforderungen erfüllen	Auswahl der Immobilie
	Robuste und wartungsarme Gestaltung der Ausstattung	Planung des Innenausbaus und Raumaufteilung (Raumplan)
		Auswahl und Umsetzung der Inneneinrichtung
		Steuerung PV-Installation und Ladesäulen-Infrastruktur etc
*IT*	Adäquate IT-Ausstattung, die Arbeiten vor Ort, remote und hybrid einfach, effizient und sicher ermöglichen	Planung, Beschaffung und Aufbau von IT-Infrastruktur
	Zufriedenheit der Mitarbeitenden	Ausrollen einer leicht nutzbaren und flexiblen Telefonie Lösung
	Nutzungsgrad der IT-Komponenten	Aufstocken der Videokonferenzmöglichkeiten
*HR*	Kulturerhalt	Mitarbeitendenbefragung
	Nähe zu den Mitarbeitenden	Aufbereitung der Anforderungen und Abstimmung mit der Leitung
	Zufriedenheit der Mitarbeitenden	
	Attraktivität als Arbeitgeber	
*Nachhaltigkeit*	Nutzung erneuerbarer Energien	Dimensionierung PV-Anlagen
	Optimierung von Verbrauchern im Einklang mit der Verfügbarkeit erneuerbarer Energien	Beschaffung E‑Bikes
		Ausbau Ladeinfrastruktur
		Einbindung eines Energiemanagementsystems

Aufgrund der unterschiedlichen Ziele und Aufgabenbereichen der Teams war ein agiles Vorgehen mit regelmäßigem, meist täglichem Austausch zwischen allen Beteiligten besonders wichtig. Für die Berichterstattung an die Geschäftsführung wurde außerdem ein sich wöchentlich treffender Lenkungskreis etabliert, in dem die Zielerreichung, der Projektplan und das Controlling diskutiert wurden. Im Folgenden wird auf die verschiedenen Beteiligten, sowie deren Ziele und Aufgaben eingegangen.

### New Work

Das New-Work-Team war, gerade zu Projektbeginn, für die Konzeptentwicklung verantwortlich. Dafür baute es die theoretische Wissensbasis zu New Work auf, nutzte Erkenntnisse der Mitarbeitendenbefragung und diskutierte Ideen regelmäßig mit den anderen Teams. Bei der Umsetzung des Konzepts stand das Team vordergründig beratend zur Seite, stellte die Erreichung des Zielbilds sicher und passte das Konzept falls nötig an neue Rahmenbedingungen an. Begleitend erarbeite das Team federführend die Kommunikationsstrategie an die Mitarbeitenden im Rahmen des Change Managements.

### Facility-Management

Das Facility-Management-Team war für die Umsetzung des ausgearbeiteten Konzepts verantwortlich. Gegenüber der „alten Welt“ hat sich dabei das Verhältnis der Raumtypen deutlich verändert. Gemessen an der Gesamtfläche nehmen offene Bereiche nun 31 % statt zuvor 16 % ein. Darüber hinaus erwies sich auch eine Standardisierung der Möblierung als Vorteil. Zum Beispiel wurden statt fest verschraubten, transportierbare Whiteboards installiert, die in jedem Büro und offenen Bereich in standardisierten Halterungen verwendet werden können. In Zusammenarbeit mit der IT kann so hohe Mobilität der Mitarbeitenden und ein unkomplizierter Wechsel zwischen Büros ermöglicht werden.

### IT

Analog dazu setzte auch das IT-Team auf Modernisierung und Standardisierung der Hardware, um die für hybride Arbeit notwendige Flexibilität und Mobilität zu gewährleisten. So wurden zum Beispiel digitale Whiteboards installiert und Kompatibilität der Endgeräte aller Mitarbeitenden mit der Infrastruktur jedes Arbeitsplatz sichergestellt. Darüber hinaus wurde die Möglichkeit einer flexiblen Zuschaltung von Konferenzteilnehmern in allen dafür vorgesehenen Räumen realisiert.

### HR

Das HR-Team agierte als Kommunikationsschnittstelle zu allen Mitarbeitenden und war für die Entwicklung eines gemeinsamen Werte- und Kulturverständnisses für die künftige Zusammenarbeit verantwortlich. Das Team führte zu Beginn des Projekts die Mitarbeitendenbefragung durch und wertete dessen Ergebnisse aus. In engem Austausch mit dem New-Work-Team und durch mehrere Workshops mit Mitarbeitenden wurde im Einklang mit geltenden Rahmenbedingungen ein individuelles Verständnis von New Work geschaffen, durch Zieldimensionen und Leitlinien konkretisiert und begleitende Maßnahmen zu deren Etablierung identifiziert.

### Nachhaltigkeit

Das Nachhaltigkeitsteam war vordergründig für die Adressierung aller ökologischen Fragestellungen rund um die Energieversorgung und das Energiemanagement verantwortlich. Dafür war es speziell mit der IT und dem Facility-Management im engen Austausch, z. B. zur Integration des Energiemanagementsystems.

Neben der Umsetzung des Konzepts ist die Erfolgsmessung, beispielsweise mit Blick auf die Akzeptanz unter den Mitarbeitenden, ein nicht zu vernachlässigender Aspekt. Hierbei stellt sich die Frage, wie Akzeptanz verlässlich gemessen werden kann. In vorliegenden Fall zeigte sich, dass während der Umsetzung Interviews und Stimmungsbilder von Mitarbeitenden aus allen Bereichen eine einfach durchführbare Möglichkeit waren. Das wiederholte Abfragen der Stimmung ist dabei wichtig, um sich ändernde Wahrnehmungen aber auch Anforderungen frühzeitig einzusammeln, um dann zielgerichtet (gegen)steuern zu können. Ein besonders wichtiger Bestandteil des Kommunikationskonzeptes war es, die Mitarbeitenden eng mit einzubeziehen und aktiv um Feedback zu werben. Dies wurde in Anspruch genommen und generiertehilfreichem Input für das Projekt. Daneben kann die Akzeptanz auch an der Nutzung der Büros quanitifiziert werden. Eine weitere Möglichkeit die Zufriedenheit der Mitarbeitenden, aber auch die Nutzung der neuen Infrastruktur zu überprüfen, ist die Auswertung der Ticketsysteme der IT und des Facility-Managements. Es zeigte sich, dass anfangs häufig noch grundlegende Anfragen aufkommen und im weiteren Verlauf das übliche Tagesgeschäft übernahm. Die einfachste Möglichkeit Erfolge hinsichtlich der Nachhaltigkeitsbemühungen zu messen, wird durch Energie(daten)managementsysteme gegeben. So können neben klassischen Kennzahlen wie dem Autarkiegrad, vermiedene CO2-Emissionen oder dem Eigenverbrauchsanteil auch detailliertere Analysen durchgeführt werden.

Um ein systematisches Reporting aufzusetzen, gibt es diverse etablierte Strategien. So thematisiert Kleindienst (2016) beispielsweise das systematische Ableiten und Messen von Kennzahlen. Hierbei legen Teams anhand der Organisationsziele eigene Teamziele fest. Diese werden in diesem Artikel in Tab. 3 exemplarisch dargestellt. Ausgehend von den Zielen können dann KPIs und deren Messung abgeleitet und operationalisiert werden.

## Good Practices

In unserem Projekt konnten auf dem Weg zur nachhaltigen hybriden Arbeit folgende Good Practices abgeleitet werden:

*Ein möglichst papierloses Arbeiten ist Grundvoraussetzung für die flexible Nutzung der Räumlichkeiten.* Nur so kann eine Mehrfachnutzung der Büroarbeitsplätze und ein flexibler Wechsel zwischen verschiedenen Raumtypen und dem Homeoffice gewährleistet werden.

*Hybride Arbeit beginnt im Kopf*. Daher muss das Bewusstsein und die Bereitschaft zu Veränderung bestehender Arbeitsstrukturen auf Führungs- und Mitarbeitendenebene geschaffen werden.

*Der Weg zur Etablierung einer hybriden Arbeitsweise ist ein Gemeinschaftsprojekt*. Die frühzeitige Einbindung aller Mitarbeitenden schafft ein gemeinschaftliches Verständnis und Grundlage der zukünftigen Zusammenarbeit. Eine gute Kommunikationsstrategie über die gesamte Transformation hinweg erhöht die Akzeptanz und sorgt für Wertschätzung unter allen Mitarbeitenden.

*Hybride Arbeit darf nicht als isoliertes Projekt betrachtet werden.* Vielmehr gilt es mittel- und langfristige Organisationsstrategien zu berücksichtigen. Gerade mit sich wandelnden Wertvorstellungen und technologischen Möglichkeiten ergeben sich kontinuierlich neue Potenziale. Als prägnantes Beispiel sind hier verschiedene Ansätze zum Thema Nachhaltigkeit zur Steigerung der Attraktivität des Arbeitsplatzes und des Arbeitgebers denkbar.

*Just go for it!* Nachhaltige hybride Arbeit wird nicht am Reisbrett theoretisch konzipiert, sondern muss gelebt und kontinuierlich weiterentwickelt werden.

## Zusammenfassung und Ausblick

Große gesellschaftliche Herausforderungen wie die COVID-19-Pandemie und der globale Klimawandel haben die Art und Weise wie Menschen zusammenarbeiten und über Arbeit nachdenken für immer verändert. In der vorliegenden Arbeit wurde die Frage adressiert, wie Arbeit gestaltet werden kann, um Vorteile der Arbeit vor Ort, Vorteile digitaler Arbeit sowie Nachhaltigkeitsaspekte nicht nur zu berücksichtigen, sondern ineinandergreifend zu integrieren.

Ausgehend vom Fall des Transformationsprojekts eines deutschen Forschungsinstituts wurden zwei Beiträge mit wertstiftenden Implikationen für Wissenschaft und Praxis geleistet. Erstens wurde ein konkretes New-Work-Konzept für nachhaltige hybride Arbeit präsentiert, das durch Erkenntnisse in der Literatur und durch Erfahrungen aus deren praktischer Anwendung entwickelt wurde. Zweitens wurden Einblicke in die Umsetzung gegeben, dabei das Zusammenspiel mehrerer interdisziplinärer Teams aufgezeigt und Good Practices auf dem Weg zu nachhaltiger hybrider Arbeit abgeleitet. Der Beitrag hebt sich von bisherigen Erkenntnissen ab, da er hybride Arbeit und Nachhaltigkeit vereint und Wege der integrierten, interdisziplinären Umsetzung aufzeigt. So geben unsere Ergebnisse anderen New-Work-Forschenden Grundlage und Struktur, um das Konzept nachhaltige hybride Arbeit und der Entwicklung und Messung ihrer Erfolgsfaktoren tiefergehend zu untersuchen. Praktiker:innen können das Konzept und die Good Practices als Blaupause für die eigene Transformation hin zu nachhaltiger hybrider Arbeit verwenden und davon ausgehend organizationsindividuelle Anpassungen vornehmen.

Auch wenn die Autor:innen als Teil des Projektteams tiefgreifende Daten über den vorliegenden Fall zur Verfügung hatten, ergeben sich bei jeder Fallstudie Limitationen hinsichtlich der Übertragbarkeit. Erstens sind die Erkenntnisse aus dem vorliegenden Fall eines anwendungsorientierten Forschungsinstituts in der Wirtschaftsinformatik nicht zwangsläufig auf andere Wirtschaftszweige und Organisationen übertragbar. Zum Beispiel weisen die Mitarbeitenden im betrachteten Fall eine im Vergleich zu anderen Organisationen überdurchschnittlich hohe Affinität für digitale Technologien auf. Auch sind die Arbeitsanforderungen an die Mitarbeitenden als Wissensarbeitende vergleichsweise homogen. Dies lässt sich nicht auf andere Organisationen übertragen, die gerade im produzierenden Gewerbe beispielsweise auch zeitlich und örtlich stärker gebundene Produktionsmitarbeitende beschäftigen. Zweitens fokussiert das präsentierte Konzept die Säule der ökologischen Nachhaltigkeit und nur nachgelagert die soziale Nachhaltigkeit. Drittens konnten bislang noch keine langfristigen Effekte hinsichtlich der Nutzung und Akzeptanz des vorgestellten Konzepts erhoben werden. Mit den oben eingeführten Aspekten zur Erfolgsmessung können weitere Erfahrungen und Daten gesammelt werden, wofür es allerdings weiterer Fallstudien bedarf.

Die letzten Jahre haben uns gezeigt, dass sich unser Verständnis von guter Zusammenarbeit durch externe Einflüsse grundlegend verändern kann. Auch wenn deswegen New Work bei den meisten Organisationen ganz oben auf der Agenda steht, bedarf es noch viele weitere Jahre an enger Zusammenarbeit zwischen Forschung und Praxis, bis wir mehr Erkenntnisse über den dafür notwendigen Wandel in der Arbeitsgestaltung gesammelt haben. Mit diesem Beitrag hoffen wir, hilfreiche Erkenntnisse und Handlungsempfehlungen für den Wandel zur nachhaltigen hybriden Arbeit zu leisten und Organisationen bei der Transformation zu unterstützen.
